# Epithelial-Macrophage Crosstalk Initiates Sterile Inflammation in Embryonic Skin

**DOI:** 10.3389/fimmu.2021.718005

**Published:** 2021-10-14

**Authors:** Oindrila Bhattacharjee, Uttkarsh Ayyangar, Ambika S. Kurbet, Vairavan Lakshmanan, Dasaradhi Palakodeti, Florent Ginhoux, Srikala Raghavan

**Affiliations:** ^1^ Centre for Inflammation and Tissue Homeostasis, Institute for Stem Cell Biology and Regenerative Medicine, Bangalore, India; ^2^ School of Chemical and Biotechnology, Sastra University, Thanjavur, India; ^3^ Integrative Chemical Biology, Institute for Stem Cell Biology and Regenerative Medicine, Bangalore, India; ^4^ Singapore Immunology Network, Agency for Science, Technology and Research, Singapore, Singapore; ^5^ Agency for Science, Technology and Research (A*STAR) Skin Research Lab (A*SRL), Singapore, Singapore

**Keywords:** macrophage, epithelia, crosstalk, ECM, sterile inflammation

## Abstract

Macrophages are highly responsive to the environmental cues and are the primary responders to tissue stress and damage. While much is known about the role of macrophages during inflammatory disease progression; the initial series of events that set up the inflammation remains less understood. In this study, we use next generation sequencing (NGS) of embryonic skin macrophages and the niche cells - skin epithelia and stroma in the epidermis specific knockout of integrin beta 1 (Itgβ1) model to uncover specific roles of each cell type and identify how these cell types communicate to initiate the sterile inflammatory response. We demonstrate that while the embryonic skin fibroblasts in the Itgβ1 knockout skin are relatively inactive, the keratinocytes and macrophages are the critical responders to the sterile inflammatory cues. The epidermis expresses damage associated molecular patterns (DAMPs), stress response genes, pro-inflammatory cytokines, and chemokines that aid in eliciting the inflammatory response. The macrophages, in-turn, respond by acquiring enhanced M2-like characteristics expressing ECM remodeling and matrisome signatures that exacerbate the basement membrane disruption. Depletion of macrophages by blocking the CSF1 receptor (CSF1R) results in improved basement membrane integrity and reduced ECM remodeling activity in the KO skin. Further, blocking the skin inflammation with celecoxib reveals that the acquired fate of macrophages in the KO skin is dependent on its interaction with the epidermal compartment through COX2 dependent cytokine production. Taken together, our study highlights a critical crosstalk between the epithelia and the dermal macrophages that shapes macrophage fate and initiates sterile inflammation in the skin. The insights gained from our study can be extrapolated to other inflammatory disorders to understand the early events that set up the disease.

## Introduction

The skin, our largest organ, protects underlying tissues from physical, chemical and pathogenic stresses and comprises of the ectoderm-derived epidermis and mesoderm-derived dermis separated by a meshwork of extracellular matrix (ECM) proteins known as the basement membrane (BM). Under physiological conditions, the epidermis constantly self-renews through resident pools of stem and progenitor cells that support the skin barrier function (1). The skin also contains a wide repertoire of immune cells both from the innate and adaptive systems that are critical for the defense and maintenance of skin homeostasis (2). In inflammatory skin diseases, such as atopic dermatitis and psoriasis, keratinocytes exacerbate inflammation by synthesizing alarmins, such as cytokines, chemokines and DAMPs which results in chronic activation of these immune cells resulting in non-resolving inflammation (3, 4). The current treatment strategies primarily focus on modulating adaptive immune responses however, much less is known about the very earliest innate immune responses that potentially drive inflammatory skin disorders in response to epidermally derived DAMPs and cytokines.

Inflammation that occurs in absence of a barrier breach is termed as sterile inflammation. Several studies have suggested that sterile inflammation is initiated primarily by the innate immune cells such as macrophages (5–7). Macrophages are a group of heterogeneous cells which exhibit remarkable plasticity. *In vitro* analysis of macrophages have revealed that they can adopt an M1 (pro-inflammatory) or M2 (pro-remodeling) polarization/activation state when exposed to different milieus of cytokines (8). Transcriptomic studies on *in vitro* primed macrophages suggest that these subtypes can be identified by distinct markers. While M1 macrophages have been shown to express proinflammatory cytokines such as *Il1b, Tnfa, Il12b* and *Ccl3* (9). M2 macrophages are identified by expression of *Arg1* (Arginase 1), *Fizz1* (resistin-like-α), *Mrc1* (CD206), *Chil3* (chitinase 3-like 3, Ym1), and *MerTK* (10). *In vivo* studies, however, suggest that the macrophages acquire a mixed signature which is, in part, a result of a large repertoire of cues received from multiple cell types in the tissue (11). These studies highlight a major gap in the understanding of how tissue macrophages integrate signals from their niches to acquire distinct functional states and how that may, in turn, influence tissue homeostasis. Since murine embryonic skin lacks a mature adaptive immune system, monocytes and macrophages make up a large part of the immune repertoire of the skin. Macrophages are recruited to the skin from the yolk sac during primitive hematopoiesis (E7.5) and from the fetal liver during definitive hematopoiesis (E13.5). Embryonic macrophages migrate and maturate into functional tissue resident cells in a CSF1R (colony stimulating factor 1 receptor) dependent manner (12, 13). Studies from our lab, and others, have shown that embryonic macrophages hold potential to actively participate in inflammatory conditions (14, 15).

Conditional knockout of Itgβ1 in the epidermis results in dermal-epidermal separation which is perceived as a wound by the embryonic skin. This results in increased macrophage recruitment into the skin that causes enhanced ECM disruption suggesting that resident embryonic skin macrophages respond actively to perceived wounds under sterile conditions (14). One of the intriguing aspects of our previous work was that while we generated an epidermal specific KO of Itgβ1, the enhanced dermal immune response elicited, suggesting a crosstalk between these two compartments. Notably, DAMPs are shown to affect macrophage fate acquisition, suggesting a plausible mechanism for the crosstalk between the epidermis and macrophages during inflammation (16–18).

In this paper we have explored the crosstalk between skin epithelia and macrophage compartment in our epidermal Itgβ1 KO model of sterile inflammation. We first establish that the resident and recruited macrophages are the most dynamic responders to the epidermal inflammatory cues in the Itgβ1 KO embryonic skin. Inflammation is an orchestrated phenomenon that involves integration of several environmental factors that impinge on macrophage effector cell functions. Therefore, to identify the relative contributions of each cell type to the inflammatory response and understand how the crosstalk between these cell types drive macrophage fates, we performed NGS analysis of the key skin components – the epidermis, fibroblasts and macrophages isolated from Itgβ1 KO skin. The NGS analysis suggests that in Itgβ1 epidermal KO embryos, the sterile inflammation is primarily driven by the epidermal and macrophage compartment while the fibroblasts are relatively inactive. The epidermis responds to the loss of Itgβ1 by acquiring a stress associated proinflammatory state resulting in the synthesis of cytokines, chemokines and DAMPs. These cytokines and chemokines further recruit and activate monocytes and macrophages. On the other hand, macrophages acquire enhanced M2-like characteristics with exaggerated ECM remodeling properties (leading to basement membrane disruption) and act as a source of an ensemble of ECM transcripts. Intriguingly, depletion of macrophages using CSF1R blocking antibodies leads to a decrease in ECM production and disruption. Likewise, blocking epidermal inflammation and cytokine release using cyclooxygenase 2 (COX2) inhibitor celecoxib leads to inhibition of enhanced M2-like fate acquisition and increased organization of the basement membrane. Taken together, our study provides evidence to suggest that sterile inflammation in skin is driven by the crosstalk between the epidermal and macrophage compartment which, in turn, dictate their cellular fates.

## Materials and Methods

### Animals

The conditional knockout of integrin beta 1 in the epidermis was generated as described by Raghavan et al. (19). Briefly, ITGB1fl/fl females were crossed with ITGB1fl/+|K14-Cre males to obtain the epidermal knockouts of integrin beta 1. Mice were housed in NCBS/inStem Animal Care Resource Centre. Animals were handled, bred and euthanized in compliance with the guidelines and procedures approved by the inStem IACUC (Institutional Animal Care and Use Committee). Animals were regularly monitored for any health concerns. All animals for experiments were housed in a specific pathogen free (SPF2) facility in ventilated cages kept under a 12-hour light and dark cycle and were given unlimited food and water. The temperature in the facility was maintained at 21°C.

### Drug Treatments

The pregnant females were intraperitoneally injected with either monoclonal CSF1R blocking antibody (AFS98) which was resuspended in 1X PBS or 1X PBS (control) on the 6th or the 7th day of the gestation and the embryos were retrieved at E17.5 and E18.5. Celecoxib (25 mg/kg resuspended in 5% DMSO) or 5% DMSO (Sigma) (control) was administered to pregnant females on the 15th, 16th, and 17th day of gestation by oral gavage as described by (14). The embryos were retrieved at E18.5.

### Immunostaining

10-micron frozen cryosections were thawed for 5 minutes at RT and fixed. Fixation was done with 4% PFA (sigma) for 10 minutes at room temperature or 100% chilled acetone (Merck) at -20C for 10-15 minutes. Slides were washed 3 times with 1X PBS for 5 minutes. This was followed by a permeabilization step with PBST (PBS with 0.2% Triton X-100 (Sigma) for membrane and cytoplasmic proteins or 0.5% Triton X-100 for nuclear proteins). Blocking was done for 40-60 minutes at room temperature, with 3% BSA (bovine serum albumin) (Himedia) or NDS (normal donkey serum) (Abcam) in PBST or PBS based on the information provided in the antibody datasheet. Tissues are outlined with a hydrophobic pen (Merck) to avoid the draining of antibodies in the following steps. Sections were incubated with one or more primary antibodies (for co-staining purposes) overnight at 4C or 1 hour at room temperature. Unbound primary antibodies were washed 3 times with 1X PBS for 5 minutes at room temperature and incubated with alexa fluor secondary antibodies (Invitrogen) in blocking buffer at a dilution of 1 in 300 for 40 min at room temperature. Unbound secondary antibodies were washed 3 times with 1X PBS for 5 minutes at room temperature. The nucleus was stained with 1X DAPI (Sigma) followed by washing with 1X PBS and mounted with mowiol. Slides were left in the open for the Mowiol^®^ 4-88 (Sigma) to dry and were then sealed with nail polish (Lakme) followed by storage at 4C. Imaging was done on the Olympus FV3000 5 LASER confocal microscope. The antibodies used were: Integrin beta 1, Fibronectin, Tenascin-C (Millipore); Laminin-332 (gifted by Bob Burgeson); Keratin-6 (gifted by Satrajit Sinha); Ki67, CD206 (Abcam); F4/80, CD3, MERTK, (Invitrogen); MMP-9 (R & D); Integrin beta 6 (gifted by Shelia Violette, Biogen Idec, Boston); Integrin beta 4 (Biolegend), Cyclooxygenase 2 (Abcam), and Arginase 1(Cell Signaling Technology).

### Hematoxylin and Eosin Staining

10 micron frozen sections were thawed for 5 min and then fixed with 4% paraformaldehyde for 10 minutes at room temperature. Slides were washed 3 times with 1X PBS for 5 minutes and stained with hematoxylin (Sigma) for 30-45 seconds. Excess hematoxylin was washed under running tap water till water becomes clear. Eosin (Sigma) (1 in 10 diluted with 70% ethanol) staining was done for 10-15 seconds. Excess eosin was removed by dipping in water. Slides were air dried and mounted with 80% glycerol (Emparta ACS) and stored at room temperature. Imaging was done on the IX 73 widefield microscope.

### Sample Preparation for FACS, RNA, and Protein Isolation

The whole skin was treated with 2mg/ml dispase (Gibco) (in 1X PBS) for 45 minutes at 37C to separate the epidermis from the dermis. The epidermis was divided into two parts: one half was stored in an RNA stabilization buffer at 4C overnight and then transferred to -80C for RNA isolation. The other half was snap-frozen in liquid nitrogen (-196C) and stored at -80C for protein extraction. The dermis was divided into three parts: One part was snap-frozen, the second part was stored in the RNA stabilization buffer (similar to the epidermis); the third part was subjected to collagenase (Sigma) (0.25mg/ml in 1X PBS) treatment for 1hr at 37C to make a single cell suspension for cell sorting experiments.

### Flow Cytometry, Sorting, and Cell Cycle Analysis

The dermal cells were strained through the 40-micron filter and washed with FACS buffer (1X PBS with 2% FBS (Gibco)) and were centrifuged at 1000 rpm for 10 minutes at 4C. All the centrifugation steps were done at 1000 rpm at 4C. The supernatant was discarded and a fresh FACS buffer was added. Cells were counted using a cell counter and equally distributed to staining tubes. Conjugated primary antibodies were added to 100ul of cell suspension and incubated for 30 minutes on ice with intermittent tapping to avoid sedimentation. The unbound antibody was washed with the FACS buffer, and the supernatant was discarded. Fresh FACS buffer was added to the tubes and the samples were taken on ice for flow cytometric analysis and sorting on the BD FACSAria fusion cell sorter. The antibodies used were: CD206(clone- C068C2; APC conjugated; Bioelgend), MERTK (clone-DS5MMER; PECY7 conjugated; Thermo Fisher) LY6C (clone-HK1.4; APC conjugated; Thermo Fisher), CD11B (clone-M1/70; APC conjugated; ebioscience) CD45 (clone-30-F11; PE conjugated; Thermo Fisher) F4/80 (clone-BM8; FITC or APC conjugated; Thermo Fisher), CD38 (clone-90; unconjugated; Abcam).

For flow analysis, the cells were gated for the live and dead using DAPI and then gated for the immune cell population using CD45, the pan immune cell marker. Either the CD45 population was further drilled down to gate for the different immune cell markers (F4/80, CD11B, and LY6C); or the live population was directly gated for immune cell markers (F4/80, CD206, CD38, and MERTK).

For cell cycle analysis, single-cell suspensions of macrophages were fixed with 4% PFA at 4C for 30 minutes that was followed by two washes with 1xPBS and centrifugation (1000 rpm, 5 minutes each). The cell pellet was resuspended in FACS buffer and stained with CD45 and F4/80 antibody as mentioned above. Next, the cells were incubated with DAPI (1mg/ml) for 30 minutes and analyzed using a BD FACSAria Fusion cell sorter. The data were analyzed using the Watson Pragmatic model on the FlowJo software.

### Real-Time PCR

Total RNA from the epidermis, fibroblasts, and the sorted F4/80 macrophages was extracted using the protocol provided with TRIzol and TRizol LS (Thermoscientific) respectively. For the RNA isolation from macrophages, 1ul of glycoblue (Ambion) was added before the precipitation of RNA using isopropanol (Merck) step, to allow visualization of the RNA pellet. RNA concentrations were measured on a nanodrop. 1g of epidermal RNA and 50ng of macrophage RNA were used to prepare cDNA first strand using the SSIII RT cDNA synthesis kit (Invitrogen). Real-time PCR was done using the SYBR green (2X) master mix (Invitrogen). The expression of mRNA was quantified by the delta CT method using 18S RNA as an endogenous control.

### Toluidine Blue Staining for Mast Cells

The protocol was adapted from (14). Briefly, the frozen sections were thawed for 5 minutes at room temperature and then fixed with 4% paraformaldehyde. The slides were then incubated with toluidine blue (Sigma) working solution for 30 seconds at room temperature. Excess dye was drained by quick washes with distilled water. Slides were mounted with DPX (Himedia).

Toluidine blue stock= 1g toluidine blue powder dissolved in 100ml of 70% of ethanol (pH 2.3).

### RNA Sequencing Analysis

Single-end RNA sequencing (1x100bp) format was performed with biological duplicates of E17.5, E18.5 (epidermis, sorted fibroblasts and macrophages) WT and KO and E17.5 CSF1R blocked (epidermis and sorted fibroblast) samples on the Hiseq 2500 using cDNA libraries. ~31 to 40 million reads were obtained for each sample. Trimmomatic adapters were used for mapping to rRNA (20). Further analyses were done with the reads that do not map to rRNA. Reference based transcriptome assembly algorithms Hisat2 v2.1.0 (21); Cufflinks v2.2.1 (22) and Cuffdiff v1.3.0 (23) were used to identify differentially expressed transcripts. The reads were aligned to mouse (mm10) genome-using Hisat2 (-q -p 8 –min-intronlen 50 – max-intronlen 250000 –dta-cufflinks –new-summary –summary-file). Cufflinks with mm10 Refseq gtf file were used to assemble the mapped reads. 4-way comparisons were done, and differential expression was calculated with Cuffdiff v1.3.0. Differentially expressed genes with adjusted p-value lesser than 0.05 and upregulated by 1.5 fold or downregulated by 0.5 were used for pathway analyses. To account for the variations between the individual replicate genes that are significantly expressed in at least two comparisons were considered. Pathway and gene-ontology analyses are performed using g-profiler (24).

### Western Blotting

Snap-frozen tissues were homogenized in liquid nitrogen with pestles and resuspended in RIPA lysis buffer with the 1X protease inhibitor cocktail added. The suspension was kept on ice and vortexed intermittently for at least 30 minutes and then centrifuged at maximum speed for 20 minutes at 4C. The supernatant containing the protein was collected and quantified by the BCA assay. 30μg of protein was loaded onto an 8% PAGE and transferred onto a PVDF membrane. Blocking was done with 5% blotto followed by overnight incubation with the primary antibodies. The next day, blots were washed with 0.1% TBST thrice and incubated with secondary antibodies for an hour at room temperature. The unbound secondary antibody was removed by washing with 0.1% TBST thrice and developed with ECL substrate. Antibodies used were: alpha-tubulin (Thermo Scientific), Cox-2(Abcam). The density of the protein bands was quantified using the Fiji software.

### Image Analysis

All image analyses were done using the Fiji software and quantified using Graphpad software.

### Statistical Analysis

All the calculations for statistical significance were performed using Graphpad software based on the data obtained from two or more biological replicates. Statistical significance was ascertained using Student’s t-test and error bars were calculated using mean with SEM.

## Results

### Myeloid Cells Form the Major Population of Immune Cells in the Integrin β1 KO Skin and Are Recruited *via* Circulation

**Figure 1:**
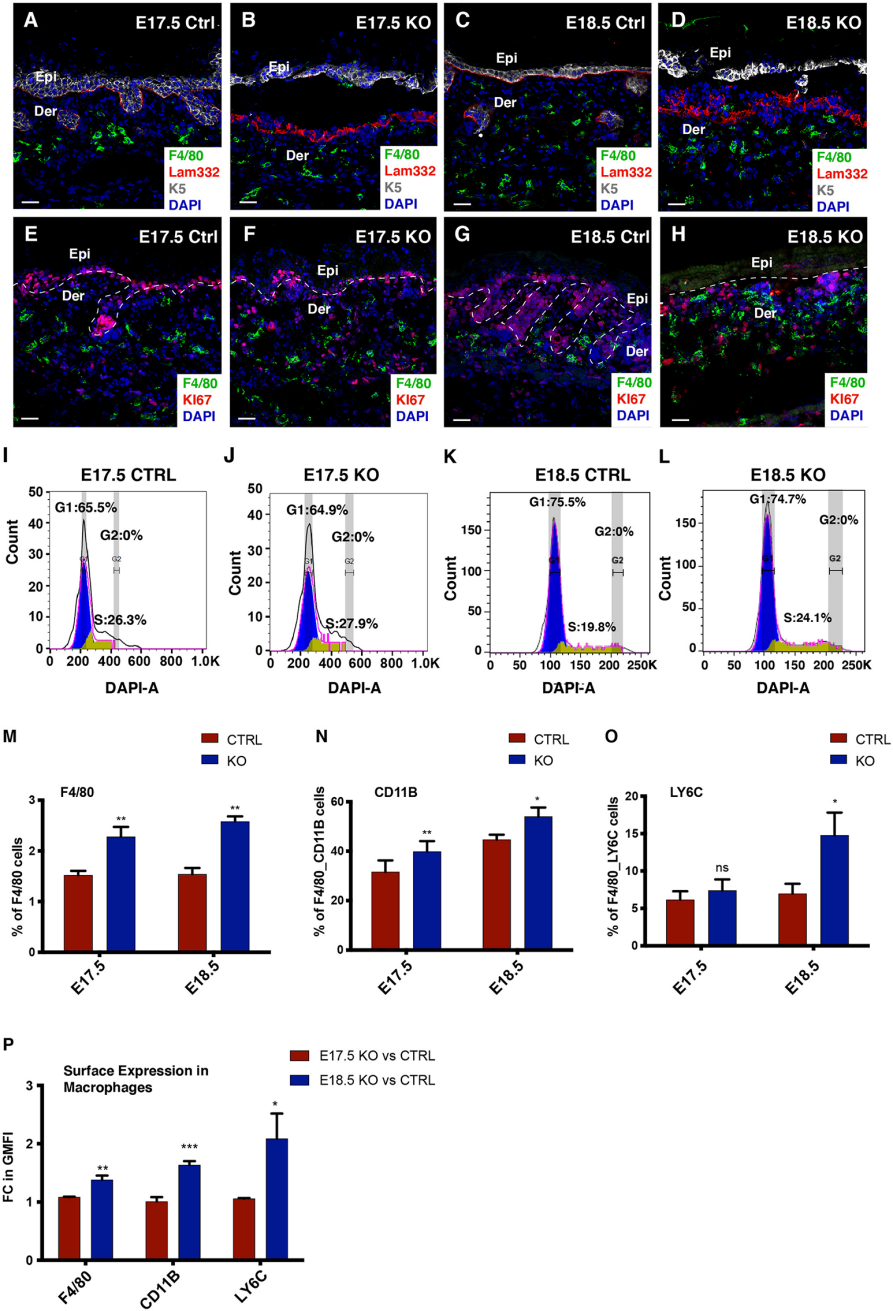
Macrophages are the major immune cells in the E18.5 skin and are derived from the circulatory monocytes. Increase in the macrophage population and BM disruption in the Itgβ1 epidermal KO skin compared to the control skin depicted by the immunostaining for F4/80, Laminin 332 (Lam-332), and Keratin 5 (K5) at E17.5 **(A, B)** and E18.5 **(C, D)**. Immunostaining of F4/80 cells with Ki67 in the control and the Itgβ1 KO skin at E17.5 **(E, F)** and E18.5 **(G, H)**. White dashed lines mark the dermal (Der)-epidermal (Epi) separation. Cell cycle analysis at E17.5 and E18.5 in the control and the KO embryonic skin **(I–L)**. Bar graphs representing percentage of F4/80 cells in the E17.5 and E18.5 control and the KO skin **(M)** (N=2). Quantification of the flow cytometry data in the E17.5 and E18.5 control and tgβ1 epidermal KO skin for the expression of F4/80^+^CD11B^+^ **(N)** and F4/80^+^LY6C^+^ **(O)** in sorted CD45^+^ cells (N=3). Quantification of the normalized geometric mean fluorescence intensity (GMFI) expression of F4/80, CD11B, and LY6C on the macrophages in the E18.5 compared to the E17.5 KOs **(P)** (N=3). Scale bars: 20 µm. *p ≤ 0.05, **p ≤ 0.01, ***p ≤ 0.001, ns=not significant.

To build a temporal understanding of the progression of sterile inflammation, we focused on embryonic stages E17.5 and E18.5. We observed disorganization of laminin 332 (a major component of the epithelial basement membrane) at the dermal-epidermal junction in the Itgβ1 epidermal KO skin which was further exacerbated by E18.5 (**Figures 1A–D**). Immunostaining and flow analysis of isolated cells from the Itgβ1 epidermal KO skin, using pan macrophage marker F4/80, suggested an enhanced macrophage burden in the KO skin compared to the littermate control (**Figures 1A–D, M**). This suggested that the progressive loss of ECM organization correlated with the enhanced macrophage numbers in the skin.

We next investigated the source of increased macrophages in the Itgβ1 epidermal KO skin. Macrophage pools in tissues can be maintained by proliferation of the existing resident macrophages or recruitment and maturation of the monocytes from circulation (25, 26). To understand if the increase in macrophage numbers was due to proliferation, we co-stained F4/80^+^ macrophages with the proliferation marker Ki67. The immunostaining data suggested that macrophages in the Itgβ1 epidermal KO skin do not proliferate as seen by the negligent nuclear Ki67 expression in F4/80^+^ macrophages (**Figures 1E–H**). This was further confirmed by the cell cycle analysis of the F4/80 macrophages (using flow cytometry) which suggested a complete absence of the G2 phase of cell proliferation in the macrophages from both control and Itgβ1 epidermal KO skin **(Figures 1I–L**). To ascertain if the increase in the macrophage number was due to the recruitment of monocytes into the Itgβ1 epidermal KO skin, we performed flow cytometry analysis for monocyte markers LY6C and CD11B. Circulating monocytes are CD11B^+^ and LY6C^+^ and acquire F4/80 expression (a macrophage marker) in the tissues (27). Flow cytometry analysis of pan CD45^+^ immune population in skin suggested a decrease in the population of CD11B^+^ and CD11B^+^F480^+^ monocytes from ~43% at E17.5 stage to ~39% at E18.5 stage in the control skin but the KO skin monocyte population increased from ~48% to ~53% (**Figure 1N** and **Supplementary Figures 1A, B, E, F**). Similar analyses for the LY6C pool suggested a slight increase in the population of LY6C^+^ and LY6C^+^F480^+^ monocytes from ~14.5% at E17.5 to ~16% at E18.5 in the control, but a significant increase in the KO skin from ~15% to ~29% (**Figure 1O** and **Supplementary Figures 1C, D, G, H**). This suggests that the increase in macrophage population in Itgβ1 epidermal KO skin was primarily due to the recruitment of circulating monocytes into the skin. Overall, the flow cytometry analysis suggested that of the total CD45^+^ population, monocytes and macrophages were the major immune cell responders in the embryonic skin at E18.5 comprising ~65% in the WT skin and ~70% in the KO skin, respectively (**Supplementary Figures 1I, J**).

Increased surface expression of CD11B, LY6C and F4/80 is associated with monocyte and macrophage activation (28–30). Consistent with this, we observed an increase in the geometric mean fluorescence intensity of F4/80, LY6C and CD11B expression in the monocytes and macrophages of E18.5 KO skin compared with E17.5 KO skin suggesting a progressive enhancement in macrophage activity (**Figure 1P**). Taken together these data suggest that the increase in macrophage pool in the Itgβ1 epidermal KO skin is driven by the recruitment of monocyte derived macrophages to the skin.

### Epidermis and Macrophages Are Key Drivers of the Sterile Inflammatory Response in Integrin β1 Epidermal KO Skin

**Figure 2:**
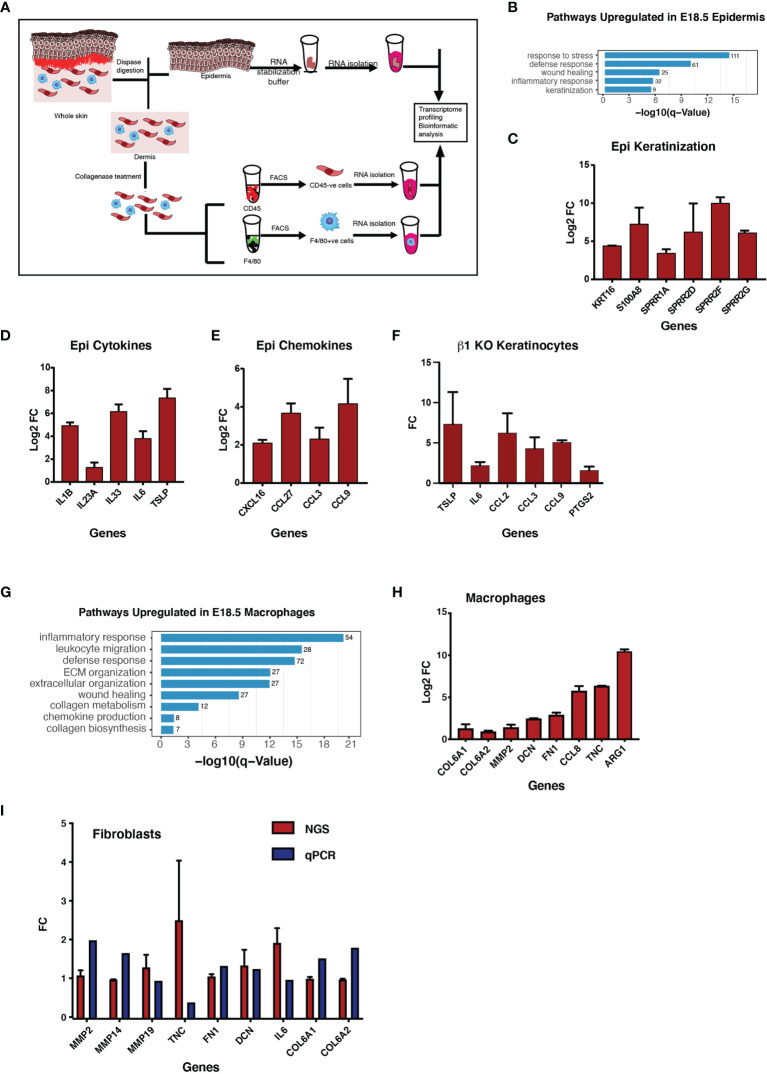
Increased expression of the proinflammatory cytokines in the epidermis and increased expression of ECM transcripts in macrophages in the KO skin. Graphical illustration of the steps involved in the isolation of epidermis, fibroblasts and macrophages to perform RNA sequencing analysis at E18.5 **(A)**. Gene Ontology analyses of pathways upregulated in the epidermis E18.5 tgβ1 epidermal KO skin **(B)** (N=2). Real-time qPCR for the cytokines, chemokines and the keratinization genes expressed in the KO epidermis at E18.5 compared to the control **(C–E)** (N=2). Fold changes in the control are normalized to 1. Real-time qPCR for the cytokines and chemokines upregulated in the β1 KO keratinocytes **(F)** (N=2). Gene Ontology analyses of pathways upregulated in the macrophages in the E18.5 KO skin **(G)**. Real-time qPCR for the ECM transcripts expressed in the macrophages in the tgβ1 epidermal KO skin compared to the control **(H)** (N=2). Fold changes in the control are normalized to 1. Bar graphs representing ECM transcripts expressed in the fibroblasts in the E18.5 KO skin **(I)** (N=2).

Inflammation is an orchestrated phenomenon consisting of several different cell types which acquire alternate functional states that, in turn, contribute towards the enhancement or suppression of inflammation. To elucidate the functional states of key cell types in the KO embryonic skin, we performed transcriptomic analysis of epidermis, dermal fibroblasts and macrophages isolated from E18.5 control and Itgβ1 epidermal KO skin using Next Generation Sequencing (NGS). To perform the NGS we isolated epidermis and dermis following dispase treatment and sorted the macrophages based on the F4/80 marker expression and isolated CD45-negative fibroblasts from the dermis as illustrated in **Figure 2A**. A comparison of cytokines, chemokines and matrisome profiles among the epidermis, fibroblasts and macrophages suggested that the epidermis and macrophages were the primary responders in the Itgβ1 epidermal KO skin (**Supplementary Table 1**). On the other hand, only minor changes in the transcriptomic profile were observed in fibroblasts derived from the Itgβ1 epidermal KO skin compared to the control skin fibroblasts suggesting that the embryonic skin fibroblasts were relatively non-responsive to the local inflammatory cues (**Figure 2I**, and **Supplementary Table 1**). We therefore omitted the analysis of fibroblasts in our experiments. The complete set of differentially expressed genes in the epidermis, macrophages, and fibroblasts is provided in the **Supplementary Table 2**.

Gene Ontology (GO) analysis of the upregulated genes in the Itgβ1 KO epidermis showed an upregulation in the pathways such as; response to stress, defense response, wound healing, and inflammatory response suggesting a role for the epidermis in orchestrating the sterile inflammation (**Figure 2B**). Detailed list of the genes belonging to these pathways are listed in the **Supplementary Figures 2A, A'**. Next, we performed real-time PCR analysis to validate some of the upregulated pathways in the NGS data. We observed an increase in the expression of keratinization associated genes *(Krt16, S100a8, Sprr1a, Sprr2d, Sprr2f, and Sprr 2g)* pro-inflammatory cytokines (*Il1b, Il23a, Il33, Il6*, and *Tslp)*, and chemokines (*Cxcl16,Ccl27, Ccl3* and*, Ccl9)* in the KO epidermis compared to the control (**Figures 2C‒E**). To investigate if the loss of Itgβ1 is sufficient for augmenting an inflammatory response in the epidermal keratinocytes, we performed real time - PCR analysis of cytokines and chemokines in the *in-vitro* cultures of Itgβ1 KO and control keratinocytes. Similar to the Itgβ1 KO epidermis, we observed an increase in the expression of cytokines and chemokines (*Tslp, Il6, Ccl2 Ccl3*, and *Ccl9*) in the KO keratinocytes compared to the control (**Figure 2F**) further highlighting the importance of Itgβ1 mediated ECM attachment in regulating epidermal homeostasis. NGS analysis of the macrophages isolated from E18.5 Itgβ1 epidermal KO skin revealed an upregulation of genes associated with GO terms that include extracellular matrix organization, collagen biosynthetic process, wound healing, and the inflammatory response (**Figure 2G**). Comparing the set of upregulated genes with the matrisome project database (31) we observed that the macrophages from Itgβ1 epidermal KO skin expressed a large repertoire of ECM transcripts that include collagens, fibronectin, and matrix remodeling enzymes with varied substrate specificities (**Supplementary Figure 2B**). We validated the expression of ECM remodeling enzymes and matrisome genes such as *Col6a1, Col6a2, Dcn, Fn1, Mmp9*, and *Tnc* in the macrophages from Itgβ1 epidermal KO skin (**Figure 2H**). Additionally, we observed an increase in the expression of CCL and CXCL family of chemokines in the NGS data obtained from the macrophages which are known to recruit immune cells from circulation (**Supplementary Table 1**).

Taken together, the NGS data revealed that in the epidermal Itgβ1 KO skin, the epidermis and macrophages are the primary responders. The epidermis acquires a pro-inflammatory state which is characterized by an enhanced expression of inflammatory cytokines, chemokines and DAMPs. These signatures are likely associated with increased recruitment and activation of monocytes and macrophages to the skin. The macrophages on the other hand acquire an exaggerated pro-remodeling state which is primarily associated with ECM synthesis and remodeling.

### Macrophages in the Itgβ1 Epidermal KO Skin Acquire M2-Like Characteristics and Are More Pro-Remodelling Compared to Their Control Littermates

**Figure 3:**
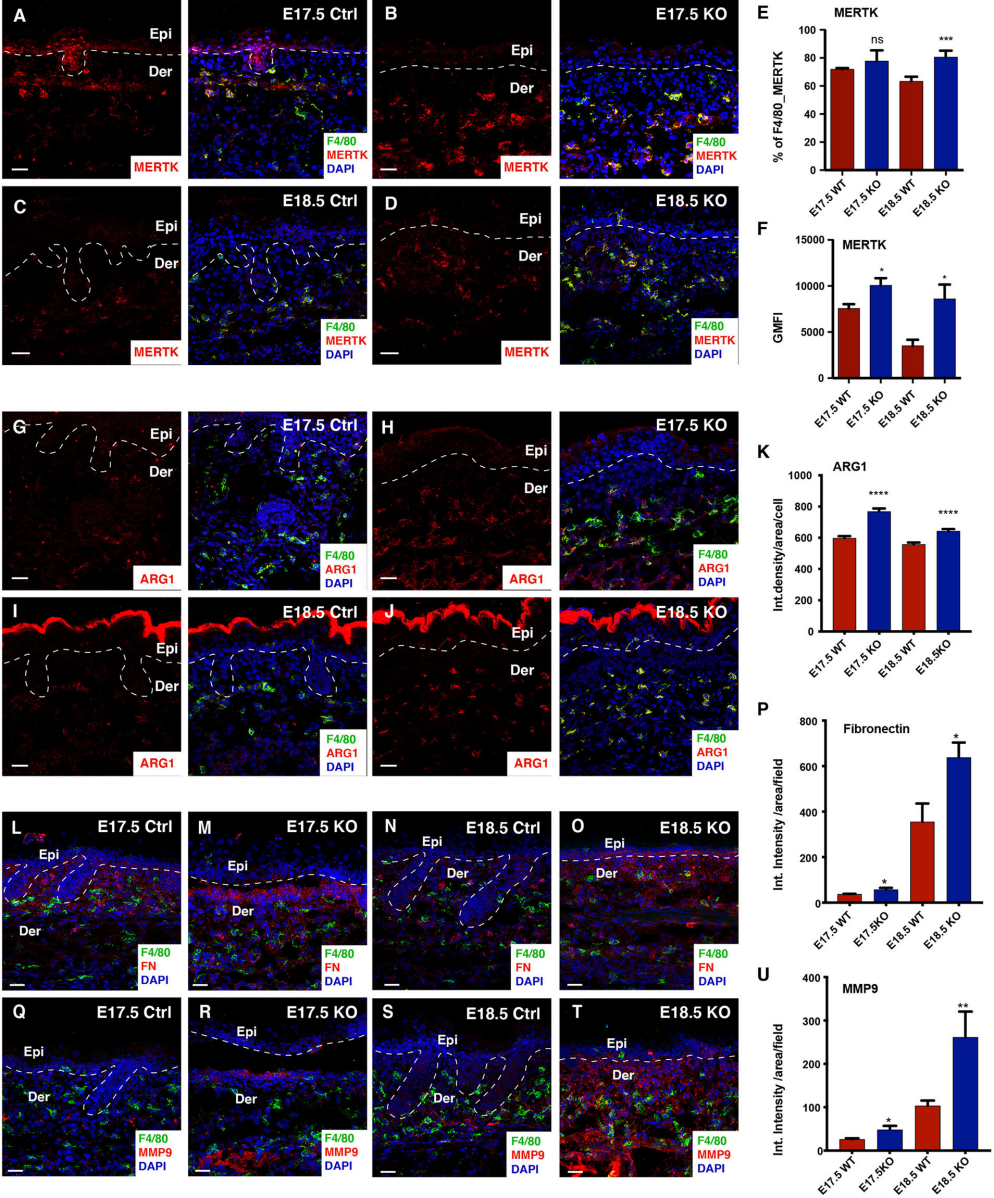
Macrophages in the KO skin acquire exaggerated M2-like pro-remodelling properties at E18.5. Immunostaining in the control and Itgβ1 epidermal KO skin at E17.5 and E18.5 for F4/80 and MERTK **(A–D)** quantified in **(E)** (N=3). Surface level expression of MERTK is quantified **(F)** (N=3). Immunostaining in the control and Itgβ1 epidermal KO skin at E17.5 and E18.5 for F4/80 and Arg1 **(G–J)** quantified in **(K)** (N=2). Immunostaining in the control and Itgβ1 epidermal KO skin at E17.5 and E18.5 for F4/80 and FN1 **(L–O)** quantified in **(P)** (N=2). Immunostaining in the control and Itgβ1 epidermal KO skin at E17.5 and E18.5 for F4/80 and MMP9 **(Q–T)** quantified in **(U)** (N=2). The white dashed line separates the epidermis (Epi) and the dermis (Der). Scale bars: 20 um. (*p ≤ 0.05, **p ≤ 0.01, ***p ≤ 0.001, ****p ≤ 0.0001, ns=not significant).

NGS data from the Itgβ1 epidermal KO skin suggested that the dermal macrophages respond to the skin inflammation by acquiring an enhanced ECM pro-remodeling state. While macrophage activation states in *in-vivo* settings have not been well characterized, *in-vitro* studies have suggested that macrophages can be broadly categorized into pro-inflammatory M1 and pro-remodelling M2 types. To understand the polarization state of macrophages from the Itgβ1 epidermal KO skin we looked at expression of distinct markers which are associated with M1 and M2 macrophage states as previously defined by Jablonski et al., 2015 (32). Our NGS analysis suggests that macrophages in the Itgβ1 epidermal KO skin express more transcripts associated with the pro-remodelling M2-like activation state compared to the pro-inflammatory M1-like signatures (**Supplementary Figure 2B**’). This includes increased expression of canonical M2-like markers such as Arginase 1 (*Arg1)*, Resistin-like molecule alpha *(Retnla)*, and Fibronectin *(Fn1)* (**Supplementary Figure 2B, B'**). To further confirm the polarization state of the macrophages we performed flow cytometry and immunostaining analysis of F4/80 macrophages with both M1 and M2 markers. Macrophages in the embryos are not yet clearly defined as M1/M2 so we wanted to understand if the macrophages in the embryonic skin express the canonical M1 (CD38) and M2 (CD206, ARG1, MERTK) markers as reported in the studies from the adults. CD38 is an ectoenzyme expressed on the surface of many cells including immune cells and is currently considered as the only well characterized marker for M1 macrophages. On the other hand, CD206, ARG1, and MERTK have been shown to be associated with ECM uptake, ECM synthesis and macrophage phagocytic activity, respectively (33–36). Immunostaining and flow cytometry analyses of macrophages from Itgβ1 epidermal KO skin revealed an increase in the population of F4/80^+^MERTK^+^ double positive macrophages and increased level of MERTK expression compared to the macrophages from control skin. (**Figures 3A–F** and **Supplementary Figures 3A–D**). Additionally, macrophages in the Itgβ1 epidermal KO skin showed an increased expression of the enzyme ARG-1 (**Figures 3G–K**). This was further associated with an increased expression of fibronectin and the matrix remodeling enzyme MMP-9 in the Itgβ1 epidermal KO skin at E17.5 and E18.5 (**Figures 3L–U**). We did not however observe significant differences in the populations of F4/80^+^CD206^+^ and F4/80^+^CD38^+^ between the control and the Itgβ1 epidermal KO skin (**Supplementary Figures 3E–R**). While we did not observe any appreciable differences in the level of MMP9 transcripts, immuno-staining data suggested an increase in MMP protein expression in the dermal compartment (**Figures 3S, T**)

Taken together, our data suggests that macrophages in the Itgβ1 epidermal KO embryonic skin are activated and acquire an enhanced M2-like pro-remodelling state.

### Macrophage Depletion Reduces BM Disruption and Epidermal Inflammation Despite Persistent Epidermal Stress

**Figure 4:**
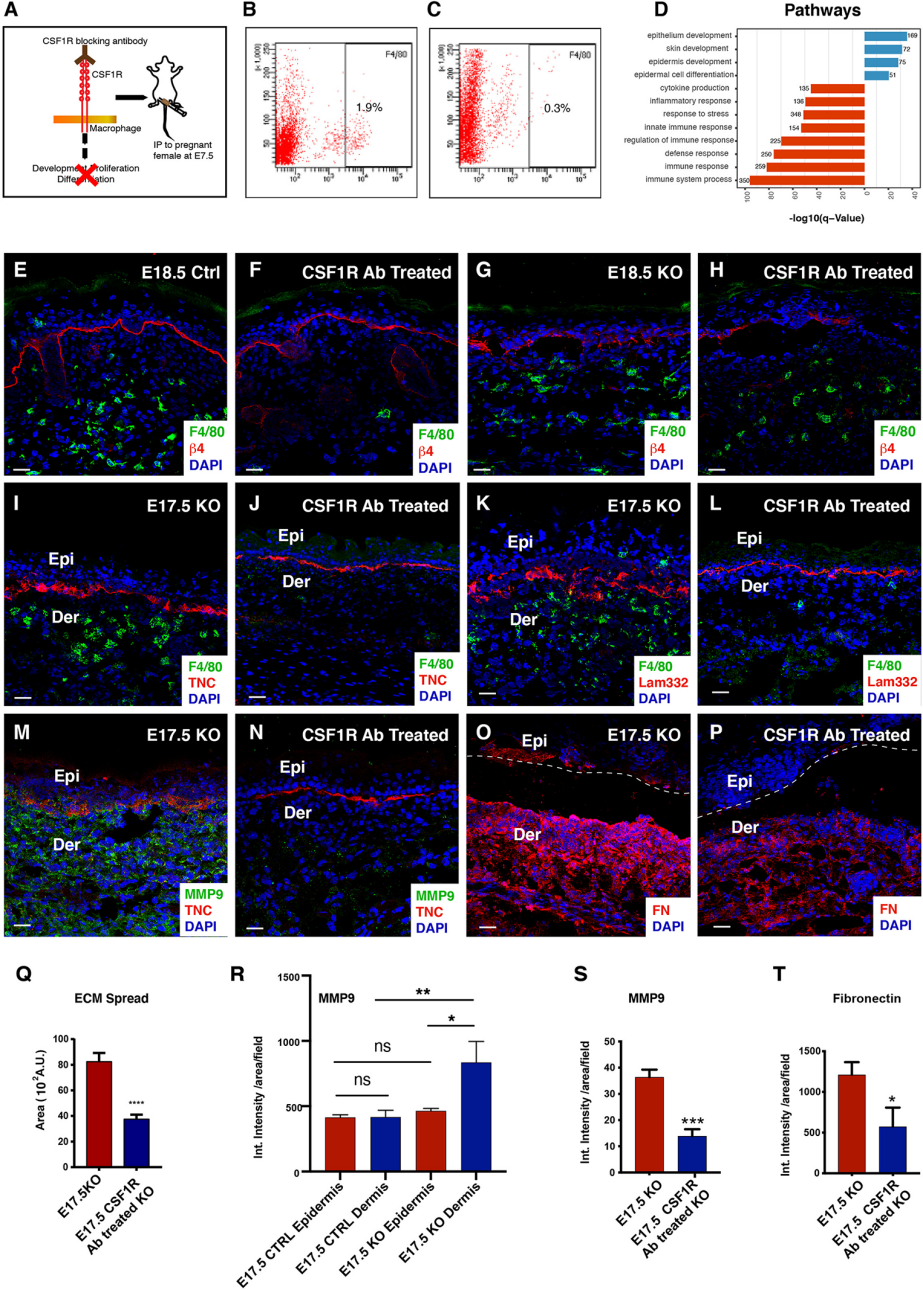
Reduced BM disruption and ECM abundance in the macrophage depleted CSF1R blocked skin. Graphical illustration of the dosing strategy of CSF1R blocking antibodies to pregnant dams **(A)**. Flow cytometry analysis for F4/80 expression in the E17.5 Epidermal Itgβ1 KO PBS and CSF1R blocked skin, respectively **(B, C)**. Gene Ontology pathway analysis for the upregulated (blue) and downregulated (red) genes in the epidermis of the CSF1R blocked KO skin **(D)**. Immunostaining for F4/80 and Itgβ4 in the control, CSF1R antibody treated, untreated Itgβ1 KO, and CSF1R antibody treated epidermis Itgβ1 KO skin at E18.5 **(E–H)**. Immunostaining in the E17.5 PBS treated and CSF1R antibody treated KO skin for F4/80 and TNC **(I, J)**; F4/80 and LAM332 **(K, L)**; MMP 9 and TNC **(M, N)** and Fibronectin **(O, P)** (N=2). The white dashed line separates the epidermis (Epi) and dermis (Der). Scale bars: 20 µm. Quantification of the area of spread of the Lam332 staining in the E17.5 epidermal Itgβ1 KO PBS and CSF1R blocked skin **(Q)** (Control N=3, CSF1R blocked N=4; ****p ≤ 0.0001). Quantification of the staining intensity of MMP9 expression in the epidermis and the dermis of the PBS and the CSF1R antibody treated KO samples **(R)** (N=2; *p ≤ 0.05, **p ≤ 0.01, ns=not significant. Quantification of the staining intensity for MMP9 and fibronectin (FN1) in the E17.5 epidermal Itgβ1 KO PBS and CSF1R blocked skin **(S, T)** (N=2; *p ≤ 0.05).

To further elucidate the pro-remodelling role of macrophages in the Itgβ1 epidermal KO skin, we depleted macrophages using a blocking antibody against the colony stimulating factor 1 receptor (CSF1R). In embryogenesis, The yolk sac and the fetal liver macrophages migrate to the developing skin starting from the embryonic day E7.5 and this migration is dependent on the CSF1R signaling (37). We, therefore, administered the CSF1R blocking antibody intraperitoneally to pregnant mice at E7.5 and analyzed the Itgβ1 epidermal KO skin from the embryos recovered at embryonic stages E17.5 and E18.5 (**Figure 4A**). In the control experiments, pregnant females were treated with PBS. Flow cytometry and immunostaining analysis of the macrophage population in the E17.5 CSF1R Ab treated KO skin suggested a remarkable decrease in the F4/80 macrophage population suggesting an effective depletion upon antibody administration (**Figures 4B, C, I, J**). However, immunostaining for F4/80 at E18.5 suggested that although CSF1R blocking persisted in the control, the effects faded in the KO (**Figures 4E–H**). Hence, to avoid ambiguity due to the waning off of antibody-mediated macrophage depletion effects by the E18.5, we focused our analysis on the E17.5 embryos. To assess the specificity of CSF1R antibody in macrophage depletion, we performed immunostaining for T cells using CD3, a pan T cell marker, and toluidine blue staining for mast cells. As expected, we did not observe differences in the T cell (data not shown) and mast cell pool between the CSF1R Ab treated and PBS treated epidermal Itgβ1 KO skin (**Supplementary Figures 4A, B, I**).

NGS data from epidermal Itgβ1 KO skin macrophages highlighted ECM synthesis and matrix remodeling roles for macrophages in the KO skin. We, therefore, investigated the impact of macrophage depletion on the status of the skin matrisome profile and the basement membrane organization. We observed a marked reduction in the disorganization of Tenascin-C (an ECM protein highly expressed during embryonic development) and Laminin-332 at the dermal epidermal junction (**Figures 4I–L, Q**). We previously reported that the loss of basement membrane organization in the epidermal Itgβ1 KO skin correlated with the enhanced activity of matrix remodeling enzymes. We therefore asked if the reduced ECM disorganization in the CSF1R blocked KO was associated with an overall reduction in matrix remodeling enzyme expression. Immunostaining based analysis of MMP9 expression suggested a marked reduction in the MMP-9 protein expression in CSF1R Ab treated KO skin compared to the control PBS treated KO skin (**Figures 4M, N, S**).

Since MMP9 is a secreted protein, we next attempted to understand which compartment in the Itgβ1 epidermal KO skin was the major contributor of this matrix remodeling enzyme. qPCR analysis on RNA isolated from the epidermis, fibroblast and macrophages from KO skin indicated that there weren’t any significant differences in the transcript levels of MMP9 in these compartments (**Supplementary Figure 3S**). We performed MMP9 immunostaining in the E17.5 and E18.5 control and Itgβ1 epidermal KO skin to quantify the expression of MMP9 protein in the epidermal and dermal compartments (**Figures 3Q–T**, **4R** and **Supplementary Figure 3T**). Our data suggested that, while MMP9 was expressed both in epidermal and dermal compartments in control skin, there was a significant increase in its expression in the E17.5 dermal compartment of the Itgβ1 epidermal KO skin (**Figure 4R**), which we also observed at E18.5; (**Supplementary Figure 3T**). These data coupled with the observation that there was a significant decrease in dermal MMP expression in KO animals treated with the CSF1R blocking Ab (**Figures 4M, N, S**) led us to conclude that dermal macrophages were the primary source of MMP9 which is associated with ECM remodeling.

Additionally, we also observed a marked reduction in the expression of the Fibronectin protein in the CSF1R Ab treated KO skin compared to the PBS KO skin (**Figures 4O, P, T**). Taken together, these observations establish macrophages as the primary source of the ECM ensemble and matrix remodeling enzymes in the Itgβ1 epidermal KO skin.

Interestingly, histological analysis of the CSF1R Ab treated KO skin suggested reappearance of the hair follicles and increase in epidermal thickness (**Supplementary Figures 4G, H, L**). This observation suggested an active response of the epidermal compartment to macrophage depletion in the dermis. To build an understanding of the epidermal response, we performed NGS analysis of the epidermis isolated from CSF1R Ab treated and the Untreated epidermis Itgβ1 KO skin at E17.5. NGS analysis from the CSF1R Ab treated KO epidermis showed an upregulation of transcripts belonging to biological processes such as; the development of the skin, epithelia, and epidermis (**Figure 4D**). Consistent with this, we observed and validated the transcriptional upregulation of epidermal differentiation complex (EDC) in the CSF1R Ab treated KO skin (**Supplementary Figure 4K**). The list of differentially expressed genes in CSF1R antibody treated KO vs untreated KO epidermis is given in **Supplementary Table 3**. As established previously, epidermis responds to the loss of Itgβ1 by upregulating the expression of proinflammatory cytokines, chemokines and stress response genes. We asked if depletion of dermal macrophages affected epidermal response to loss of Itgβ1. qPCR analysis of the CSF1R Ab treated showed a significant reduction in the expression of pro-inflammatory cytokines, such as *Il1b, Il6, Il12b*, and *Il23a* and chemokines involved in T cell recruitment such as *Cxcl10 and Cxcl16* compared to PBS treated KO skin(**Supplementary Figures 4M, N**). Interestingly, we observed a significant increase in the macrophage recruiting chemokine *Ccl2* (**Supplementary Figure 4N**). This suggests that the system responds to the absence of macrophages in the dermis by upregulating macrophage recruiting signals. On the other hand, we observed no change in the expression of epidermal stress response genes and DAMPs as seen by qPCR analysis (**Supplementary Figure 4J**). To further assess the status of epidermal stress we analyzed the expression of Itgβ6 and KRT-6. We previously reported that the loss of Itgβ1 from the epidermis led to increased expression of KRT-6 (a stress associated keratin protein), and *de novo* expression of integrin beta 6 (Itgβ6), a wound response integrin (14). The expression of KRT-6 and Itgβ6 persisted in the CSF1R Ab treated KO skin (**Supplementary Figures 4C–F**) suggesting that while the epidermal stress response was primarily due to the loss of Itgβ1; the cytokine and chemokine profile was modulated by the dermal macrophages in the KO skin.

Taken together, the macrophage depletion experiments provide proof of the concept that macrophages in the KO skin regulate ECM organization by directly acting as a source of ECM ensemble and matrix remodeling enzymes. Several studies have established the role of cytokines in polarizing macrophages to a pro-remodelling M2 fate (9, 38).

### Epidermal Inflammation Prime Pro-Remodeling Fate Acquisition in the KO Macrophages

**Figure 5:**
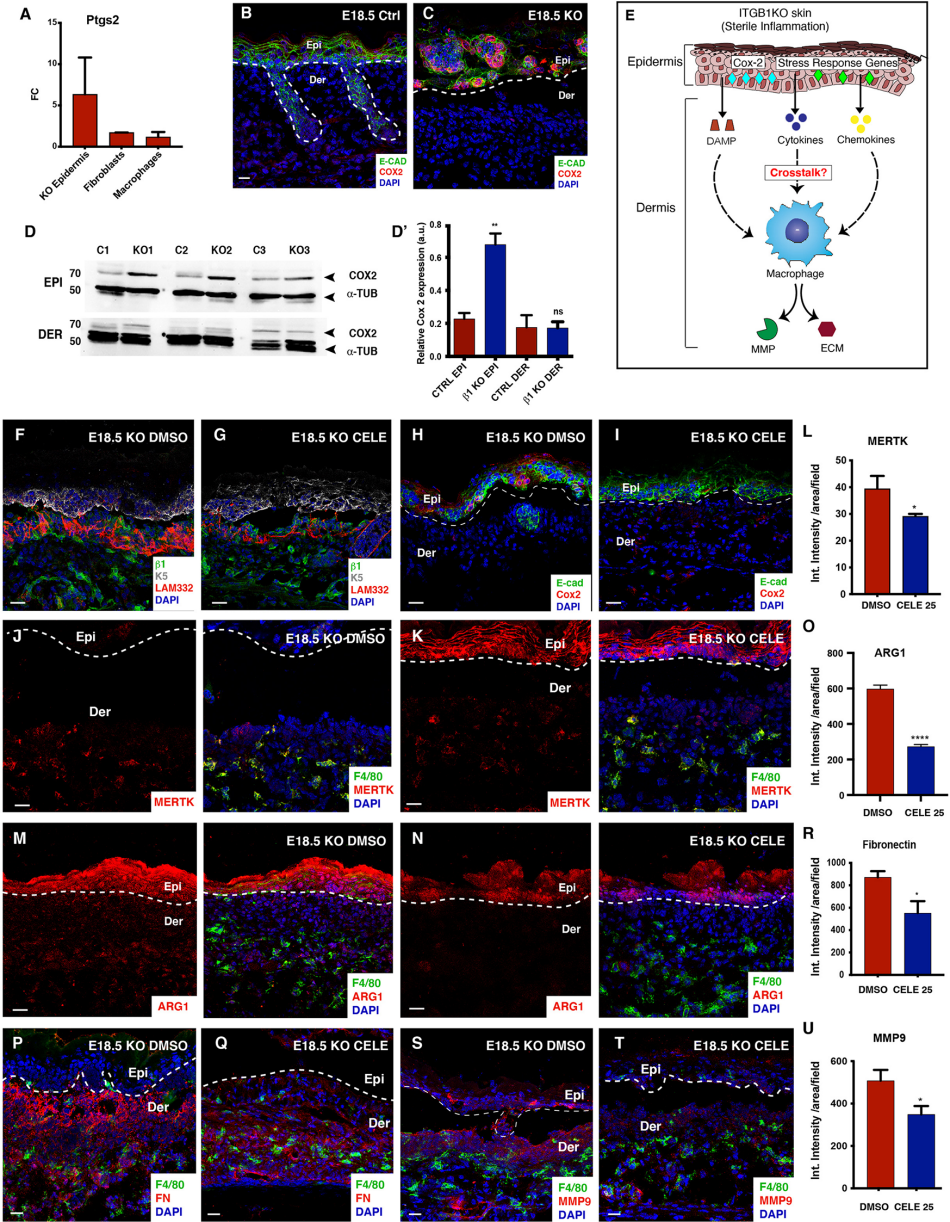
Reduced BM remodeling and ECM abundance in the celecoxib treated E18.5 KO skin. Quantification of *Ptgs2* transcript expression in the epidermis, fibroblasts and macrophages in the E18.5 Itgβ1 epidermal KO skin compared to the control **(A)**. Fold changes in the control are normalized to 1 (N=2). Immunostaining for COX2 and E-cadherin (Ecad) expression in the E18.5 control and the Itgβ1 epidermal KO skin **(B, C)** (N=2). Western blot of COX-2 in epidermis and dermis from Control and KO skin at E18.5 **(D)** which is quantified **(D')**. α-tubulin is used as endogenous control. 70 and 50 represent molecular weight of proteins in KDa. Graphical representation of COX-2 driven epithelial-macrophage crosstalk in the epidermal Itgβ1 KO skin **(E)**. Immunostaining in the E18.5 KO epidermis treated with DMSO and celecoxib for LAM332, Itgβ1, and Keratin 5 (K5) **(F, G)**; COX2 and Ecad **(H, I)** (N=2). Immunostaining for F4/80 and MERTK **(J, K)** quantified by flow cytometry in **(L)** (N=3). Immunostaining for F4/80 and Arginase 1 (Arg1), **(M, N)** quantified in **(O)**. Immunostaining for F4/80 and Fn1 **(P, Q)** quantified in **(R)**. Immunostaining for F4/80 and MMP9 **(S, T)** quantified in **(U)**. The white dashed line separates the epidermis (Epi) and the dermis (Der). Scale bars: 20 µm. *p ≤ 0.05, **p ≤ 0.01, ****p ≤ 0.0001, ns, not significant.

Next, we wanted to gain insights about the underlying mechanisms that primes the macrophages to acquire the pro-remodelling fate. As previously established in this study, the Itgβ1 epidermal KO epidermis expressed increased levels of cytokines and chemokines. The production of the inflammatory cytokines is mediated by Cyclooxygenase-2 (COX2) (*ptgs2*) which belongs to the family of prostaglandin endoperoxide synthases and is involved in the production of prostaglandins from arachidonic acid. We next aimed to understand if increased expression of the cytokines in the Itgβ1 KO epidermis was associated with an increased Ptgs2 expression. NGS, immunostaining, and the western blotting analysis suggested a significant increase in the Cox-2 expression primarily in the KO epidermis (**Figures 5A–D**’). To establish if the loss of Itgβ1 was sufficient to upregulate the expression Ptgs2, we performed qPCRs on RNA isolated from cultured control and KO keratinocytes and observed an increase in the *Ptgs2* transcript levels in the KO (**Figure 2F**).

Several studies have established the role of cytokines and DAMPs in polarizing macrophages to a pro-remodelling M2 fate (38). We therefore hypothesized that the cytokines and DAMPs produced from the epidermal compartment might regulate macrophage recruitment and activation to an M2-like pro-remodelling state in the Itgβ1 epidermal KO skin (**Figure 5E**). To test this hypothesis, we dosed pregnant females with the COX-2 inhibitor, celecoxib (25mg/kg by oral gavage) at day E15.5, E16.5 and the E17.5 of gestation (**Supplementary Figure 5A**). Pregnant females were also treated with DMSO as a control. Since COX2 is primarily expressed in the epidermis, we concluded that celecoxib targets the epidermal compartment (**Figures 5A–D**’). As expected, qPCR and immunostaining analysis of the celecoxib treated epidermis suggested a significant reduction in the expression of the Ptgs2 that was associated with a reduction in the transcripts of the inflammatory cytokines *Il6, Il1b*, and *Il23* (**Figures 5H, I** and **Supplementary Figures 5H, I**). However, we did not observe significant changes in the epidermal stress response genes and macrophage recruiting chemokines (**Supplementary Figures 5J, K**). Consistently, we did not observe a significant decrease in the F4/80+ve macrophages, F4/80^+^CD11B^+^, and F4/80^+^LY6C^+^ population. Instead, we observed a reduction in the surface expression of F4/80 on the macrophages in the celecoxib treated KO skin. This suggested that celecoxib might regulate the activation state of macrophages (**Supplementary Figures 5B–F**).

As previously described in the study, macrophage activation is associated with enhanced ECM remodeling. We observed a significant reduction in the disorganization of Lam-332 and the re-appearance of hair placodes (**Figures 5F, G** and **Supplementary Figures 5G**) associated with the reduction in the MMP-9 expression (**Figures 5S–U**). Since ECM disorganization and MMP9 expression are associated with M2-like polarization of macrophages, we asked if celecoxib treatment inhibited the expression of canonical M2 markers on the macrophages in the Itgβ1 epidermal KO skin. Immunostaining analysis of macrophages in the celecoxib treated KO skin showed reduced expression of MERTK and Arg1 compared to the DMSO treated KOs (**Figures 5J–O**). We also observed a reduction in the expression of Fibronectin in the celecoxib treated skin (**Figures 5P–R**). To specifically elucidate the impact of celecoxib treatment on macrophage pro-remodeling phenotype we performed qPCR analysis of FACS sorted macrophages isolated from DMSO and celecoxib treated skin. Our data revealed a significant reduction in the expression of ECM remodeling enzymes and matrisome genes (**Supplementary Figure 5L**). These data suggested that inhibition of epidermal COX2 attenuated M2-like pro-remodelling fate acquisition in the dermal macrophages.

**Figure 6:**
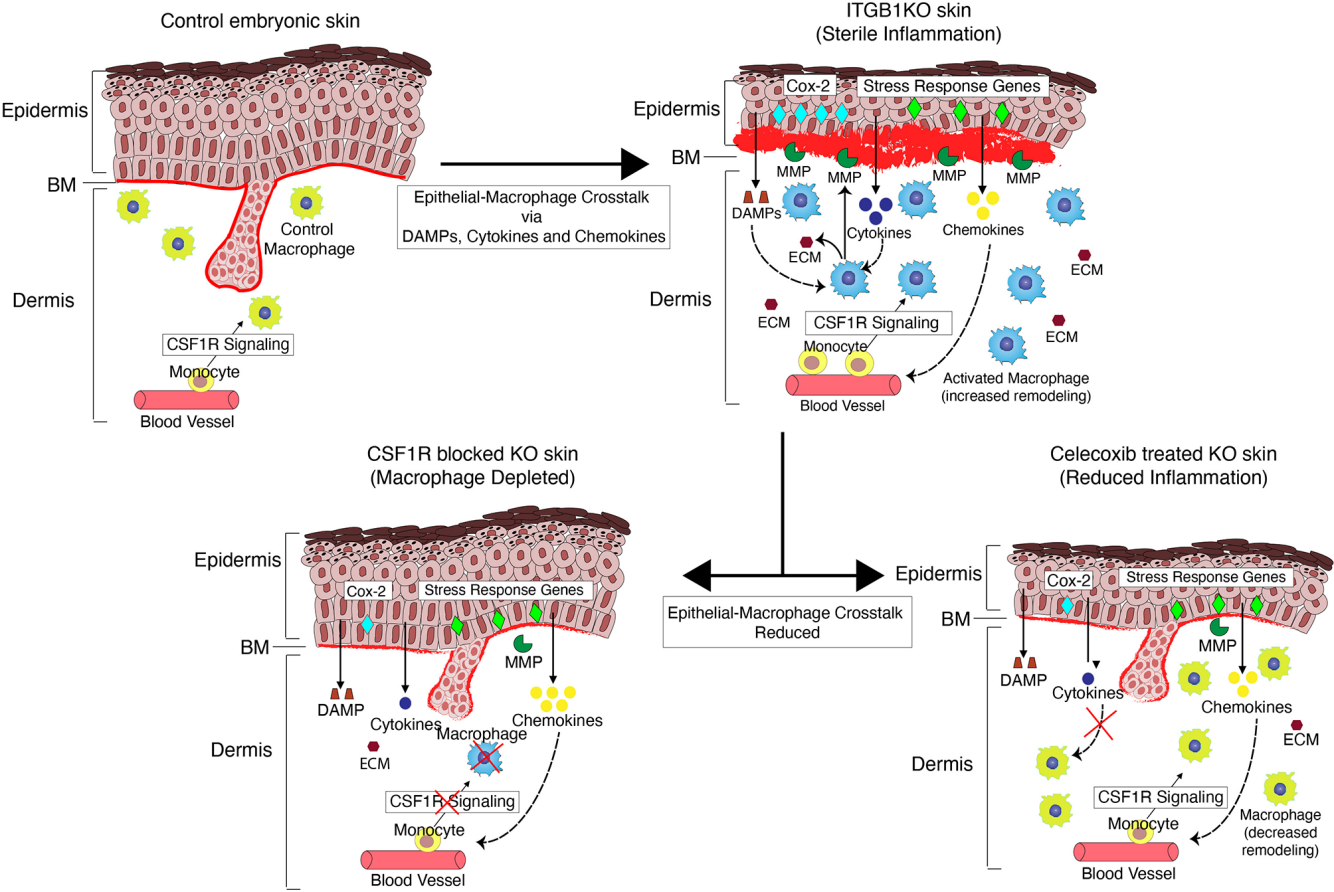
A qualitative model illustrating the epithelial-macrophage crosstalk in the Itgβ1 KO skin.

Overall, our data show that cytokines derived from the Itgβ1 KO epidermis polarize dermal macrophages to an enhanced M2-like pro remodeling fates and this epidermal-macrophage crosstalk, in turn, exacerbates the ECM remodeling in Itgβ1 epidermal KO skin (**Figure 6**).

## Discussion

We show that the sterile inflammation in the Itgβ1 KO skin is primarily driven by the epithelial and macrophage compartment. Our study provides insights into the role of macrophages during embryonic inflammation and the influence of inflammatory signals from the epidermis on the pro-remodeling properties of the macrophages. The skin epithelia responds to the loss of Itgβ1 by upregulating the expression of cytokines, chemokines and DAMPs. The expression of COX2 in the epithelial compartment results in an increased production of cytokines that activate tissue resident and monocyte derived macrophages. The macrophages respond to these epithelial inflammatory signatures by acquiring an enhanced M2-like pro-remodelling phenotype that results in increased ECM synthesis and production of matrix remodeling enzymes resulting in exacerbated basement membrane disruption. Consistently, macrophage depletion leads to reduced basement membrane disruption and ECM synthesis. We show that the acquisition of the enhanced M2-like properties of macrophages is primed by the epidermally derived cytokines as blocking the skin inflammation with celecoxib, leads to a reduction in ECM production and basement membrane disruption. Taken together, our study highlights the importance of the epithelial-immune crosstalk in priming cell fate switches in macrophages that initiate and drive sterile inflammation in the developing embryonic skin.

Inflammatory responses involve several different tissue resident cell types that contribute towards augmenting or suppressing inflammation. These distinct cell types directly influence the functional output of the highly plastic resident and recruited immune cells through their characteristic secretome (39, 40). Between the myeloid and the lymphoid immune cell repertoire, the myeloid cells are the primary responders to the inflammatory cues in tissues that eventually prime the adaptive immune response (41–43). The adaptive system further directs myeloid cell function and in turn, dictate disease outcome (44–46). While several studies have focused on understanding the role of lymphoid cells in exacerbating inflammatory disease conditions, much less is understood about the earliest interactions between the myeloid and the niche cells that initiate these inflammatory responses. The Itgβ1 KO sterile inflammatory model provides us with an excellent opportunity to begin to uncover the earliest interactions between the macrophages and niche cells.

Our study suggests that embryonic skin resident macrophages acquire an enhanced M2-like pro-remodelling properties which is also observed in other sterile inflammatory conditions such as cancer (47). Interestingly, fibroblasts in the adult skin respond to inflammatory conditions such as wounds by getting activated and secreting ECM components such as collagen, which aids in wound contraction (48). While several reports have suggested that macrophage-derived factors regulate enhanced ECM generation by the fibroblasts there is a paucity of evidence from both *in vivo* and *in vitro* studies that attribute macrophages as a direct source of the ECM (49–51). Interestingly, our data suggest that the embryonic fibroblasts are relatively inert to the inflammatory cues and that much of the ECM remodeling role is regulated by the M2-like pro-remodelling macrophages.

The M2 polarization state acts as a double-edged sword. While M2 macrophages have been shown to suppress inflammation and promote tissue homeostasis, chronic M2 activation is associated with the increased production of pro-fibrotic mediators and thus exacerbated inflammation in disorders such as the lung fibrosis (52, 53). They also play a direct causative role in autoimmune disorders such as Systemic Lupus Erythematosus (SLE) and systemic juvenile idiopathic arthritis (54). In a contact dermatitis mouse model, macrophages were shown to exacerbate inflammation by secreting factors such as IL1β, IL6 and MMP12 (55). In many cancers, tumor associated macrophages (TAM) acquire an M2-like fate that promotes metastasis by enhancing angiogenesis and ECM remodeling (56, 57). Given the strong correlation between the macrophage fate acquisition and the disease progression, there is increasing focus on understanding the critical mediators that drive macrophage polarization states in order to develop targets for therapeutic interventions (58). For example, COX2 is required for the polarization of macrophages to an M2 state and several studies have reported that blocking the COX2 mediated inflammation using celecoxib treatment improves disease prognosis in the diseases discussed (59, 60). Additionally, recent single cell transcriptomic studies suggest that macrophages and niche cells acquire a phenotype similar to their fetal counterparts in cancer and other inflammatory skin conditions in adults (39, 61). These studies highlight the potential of studying the embryonic immune system which can serve as a proxy to understanding innate immune cell dynamics that drive early stages of inflammatory diseases in adults.

Several reports have highlighted the critical role of inflammatory cytokines in driving sterile inflammatory responses in disease conditions such as cancer, arthritis and fibrosis as well as inflammatory skin conditions such as atopic dermatitis and psoriasis (62–64). Under these conditions, the epidermal keratinocytes have been shown to be an important source for inflammatory cytokines which is associated with the increased expression of COX2 (65, 66). Additionally, in these disease conditions, immune cells have been shown to acquire alternative fates which further drive the inflammatory response (67, 68). Specifically, in cancer and arthritis, macrophages have been shown to be associated with enhanced ECM remodeling activity which drives disease progression (69, 70). While inhibition of inflammation using anti-inflammatory drugs and monoclonal antibodies against cytokines have been shown to improve disease prognosis much less is understood about the effect of these anti-inflammatory drugs on immune cell fates (71, 72). Our study illustrates that, in sterile inflammatory conditions, epidermal keratinocyte derived inflammatory cytokines prime enhanced M2 polarization that, in turn, exacerbates ECM disruption. Interestingly, by reducing the inflammation using celecoxib, we could also rescue the basement membrane defects. Therefore, our study provides evidence that anti-inflammatory drugs target the cytokine mediated crosstalk between the niche cells and macrophages in sterile inflammatory conditions.

Taken together, our work summarizes the fundamentals of crosstalk between the epithelia and the innate immune compartment in setting up inflammation in the embryonic skin. The insights gained from our study can be extrapolated to other inflammatory disorders to understand the early events that set up the disease. These lines of evidence could open up a more precise crosstalk driven therapeutic targeting of inflammatory disease conditions.

## Data Availability Statement

The datasets presented in this study can be found in online repositories. The names of the repository/repositories and accession number(s) can be found below: The NGS files were uploaded at NCBI with ID: SRP324814 (PRJNA739149). The data can also be accessed using the link: https://dataview.ncbi.nlm.nih.gov/object/PRJNA739149?reviewer=1klobv7agr78somt2toldvkjvu.

## Ethics Statement

The animal study was reviewed and approved by inStem Institutional Animal Ethics Committee. Mice were housed in NCBS/inStem Animal Care Resource Centre. Animals were handled, bred and euthanized in compliance with the guidelines and procedures approved by the inStem IACUC (Institutional Animal Care and Use Committee). Animals were regularly monitored for any health concerns. All animals for experiments were housed in a specific pathogen free (SPF2) facility in ventilated cages kept under a 12-hour light and dark cycle and were given unlimited food and water. The temperature in the facility was maintained at 21°C.

## Author Contributions

SR, OB, and UA conceived the manuscript. OB performed the flow cytometry and sorting experiments. OB and UA performed the rest of the experiments, analysis, and quantifications. ASK helped with confocal imaging. VL and DP analyzed the NGS data. FG provided the CSF1R blocking antibody. SR, OB, UA, and ASK wrote the manuscript.

## Conflict of Interest

The authors declare that the research was conducted in the absence of any commercial or financial relationships that could be construed as a potential conflict of interest.

## Publisher’s Note

All claims expressed in this article are solely those of the authors and do not necessarily represent those of their affiliated organizations, or those of the publisher, the editors and the reviewers. Any product that may be evaluated in this article, or claim that may be made by its manufacturer, is not guaranteed or endorsed by the publisher.

## Supplementary Material

Supplementary Figure 1 Increased macrophage burden in the E17.5 KO skin is due to recruitment of monocyte-derived macrophages from the circulation and not proliferation. Flow cytometry analysis showing the gating strategy for the immune cell populations using CD45 as a pan immune cell marker in the control and Itgβ1 epidermal KO skin for the expression of F4/80 and CD11B, F4/80 and LY6C at E17.5 and E18.5 **(A–H)**. Pie-charts representing the total myeloid cell percentages in the E17.5 and E18.5 control and epidermal Itgβ1 KO skin **(I, J)**.

Supplementary Figure 2 Genes expressed in the epidermis, fibroblasts and the macrophages in the KO skin. Heat map represents the NGS analysis of the upregulated genes in the stress, defense, wound healing and the keratinization pathways in the Itgβ1 KO epidermis **(A, A’)**. ECM transcripts upregulated in the macrophages in the Itgβ1 KO skin **(B)**. Pro-inflammatory and the pro-remodelling associated genes in the macrophages in the epidermal Itgβ1 KO skin **(B’)**.

Supplementary Figure 3 Macrophages in the KO skin acquire exaggerated M2-like pro-remodelling properties at E17.5. Flow cytometry analysis in the E17.5, E18.5 control and Itgβ1 KO skin for the expression of F4/80 and MERTK **(A–D)** F4/80 and CD206 **(E–H)** F4/80 and CD38 **(I–L)**. Immunostaining for F4/80 and CD206 at E17.5, E18.5 **(M–P)**. Scale bar: 20 µm. Quantification of flow cytometry analysis for the percentage of F4/80^+^CD206^+^ and F4/80^+^CD38^+^ cell population in the skin at E17.5 and E18.5 **(Q, R)**. Quantification of real-time PCR analysis of the MMP9 transcript in the epidermis, fibroblasts and macrophages at E18.5 **(S)**. Quantification of the staining intensity of the MMP9 in the epidermis and the dermis at E18.5 in Control and Itgβ1 epidermal KO skin **(T)** (N=2; ***p≤0.001, ns=not significant).

Supplementary Figure 4 Epidermal stress persists in the CSF1R blocked E17.5 KO skin. Toluidine blue assay for the mast cells in the PBS treated E17.5 Itgβ1 epidermal KO skin and CSF1R antibody treated skin **(A, B)** quantified in **(I)**. Immunostaining for Keratin 6 (K6) and Itgβ4 **(C, D)**; Itgβ6 and TNC **(E, F)** PBS treated E17.5 Itgβ1 epidermal KO skin and CSF1R blocked skin Scale bar: 20 µm. Hematoxylin and eosin staining in the PBS treated E17.5 Itgβ1 epidermal KO skin and CSF1R blocked skin **(G, H)**. Scale bar: 50 µm. Quantification of the real-time PCR data for stress response and epidermal differentiation complex genes in the PBS treated E17.5 Itgβ1 epidermal KO skin and CSF1R antibody treated skin **(J, K)** (N=3; *p-value ≤ 0.05, **p-value ≤ 0.01, ns, non-significant). Quantification of the epidermal thickness in the PBS treated E17.5 Itgβ1 epidermal KO skin and CSF1R antibody treated skin **(L)** (N=3; **p-value<0.01). Quantification of the real-time PCR data in and CSF1R antibody treated skin for the cytokines and chemokines **(M, N)** (N=3; *p-value ≤ 0.05, **p-value ≤ 0.01, ****p-value≤ 0.0001 ns, non-significant).

Supplementary Figure 5 Analysis of celecoxib treated epidermis and macrophages. The representation of the strategy of dosing celecoxib to pregnant dams **(A)**. Flow cytometry analyses for the percentage of F4/80^+^, CD11B^+^ and LY6C^+^ on the CD45^+^ cells in the E18.5 Itgβ1 epidermal KO skin treated with DMSO and celecoxib **(B)**. Quantification of the changes in the percentage of population of F4/80^+^, CD11B^+^ and LY6C^+^ cells **(C, E, F)** (N=2). Quantification of the surface expression of F4/80 **(D)** (N=2). Quantification for the ECM spread in the E18.5 Itgβ1 epidermal KO skin treated with DMSO and celecoxib **(G)** (N=2; ****p-value ≤ 0.0001). Quantification for the epidermal expression of Cox2 in the E18.5 Itgβ1 epidermal KO skin treated with DMSO and celecoxib **(H)** (N=2; ****p-value ≤ 0.001). Quantification of the real-time PCR data for the cytokines, chemokines, and stress response genes in the epidermis of E18.5 Itgβ1 KO treated with DMSO and celecoxib **(I–K)** (N=3; *p-value ≤ 0.05, **p-value ≤ 0.01, ****p-value≤ 0.0001 ns, non-significant). Quantification of real-time PCR analysis for matrisome transcripts in the macrophages in E18.5 Itgβ1 epidermal KO skin treated with DMSO and celecoxib **(L)** (N=3; *p-value ≤ 0.05).

Supplementary Table 1 Expression of cytokines, chemokines and matrisome in the epidermis, fibroblasts and macrophages. Fold changes observed in the NGS analysis for the cytokines and chemokines and matrisome transcripts in the Itgβ1 epidermal KO skin compartments: epidermis, fibroblasts and macrophages at E18.5.

Supplementary Table 2 List of differentially expressed genes with FDR<0.05 in the epidermis, fibroblasts, and the macrophages of E18.5 epidermal Itgβ1 KO skin.

Supplementary Table 3 List of differentially expressed genes with FDR<0.05 in the epidermis of the CSF1R antibody treated epidermal Itgβ1 KO skin at E17.5.
