# Supplementary figures and images for: Epithelial-Macrophage Crosstalk Initiates Sterile Inflammation in Embryonic Skin
Source: Front Immunol. 2021 Oct 14;12:718005. doi: 10.3389/fimmu.2021.718005 (PMC8553113; doi:10.3389/fimmu.2021.718005)

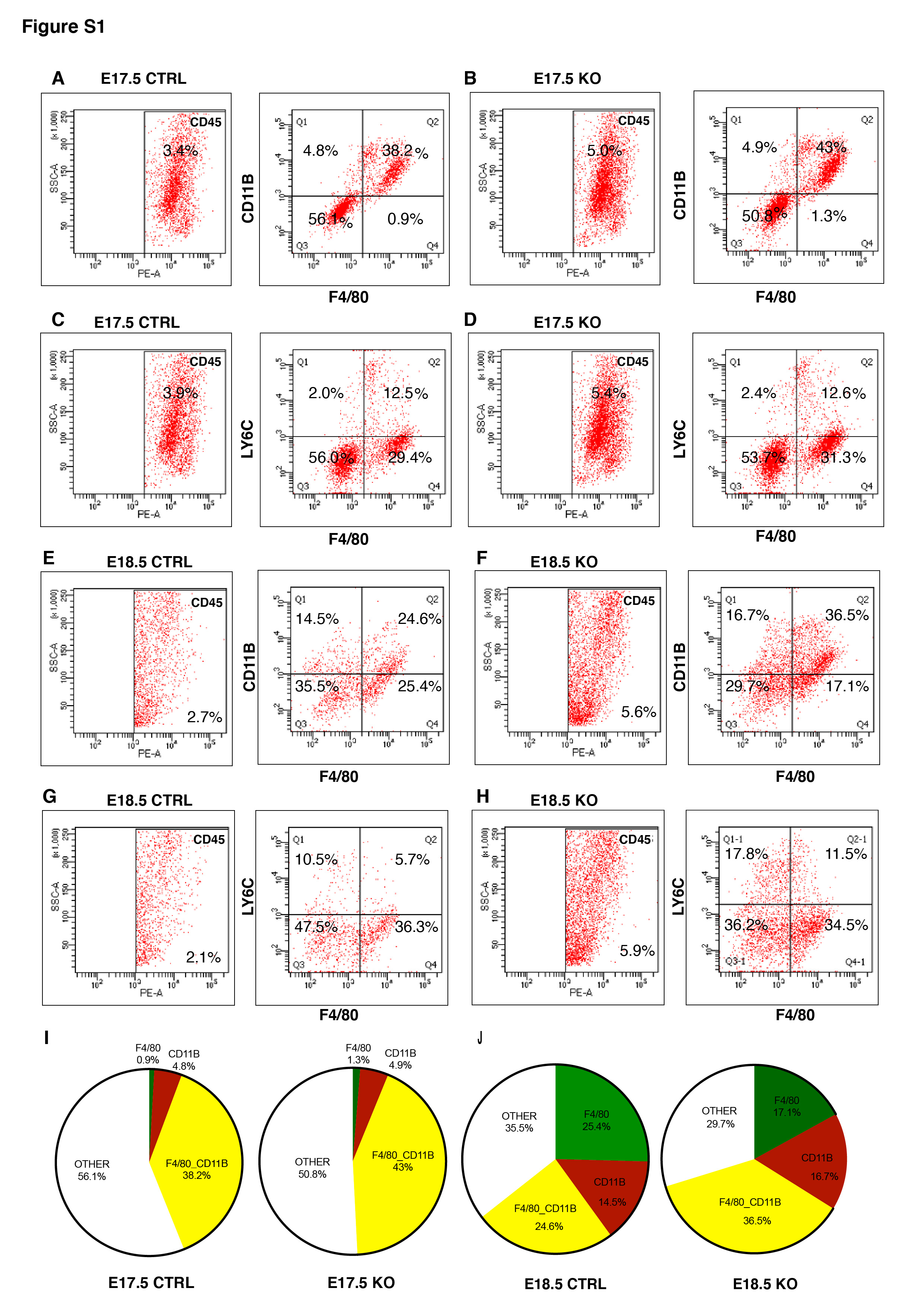

Supplement: Supplementary Figure 1 — Increased macrophage burden in the E17.5 KO skin is due to recruitment of monocyte-derived macrophages from the circulation and not proliferation. Flow cytometry analysis showing the gating strategy for the immune cell populations using CD45 as a pan immune cell marker in the control and Itgβ1 epidermal KO skin for the expression of F4/80 and CD11B, F4/80 and LY6C at E17.5 and E18.5 (A–H). Pie-charts representing the total myeloid cell percentages in the E17.5 and E18.5 control and epidermal Itgβ1 KO skin (I, J). [file Image_1.jpeg]

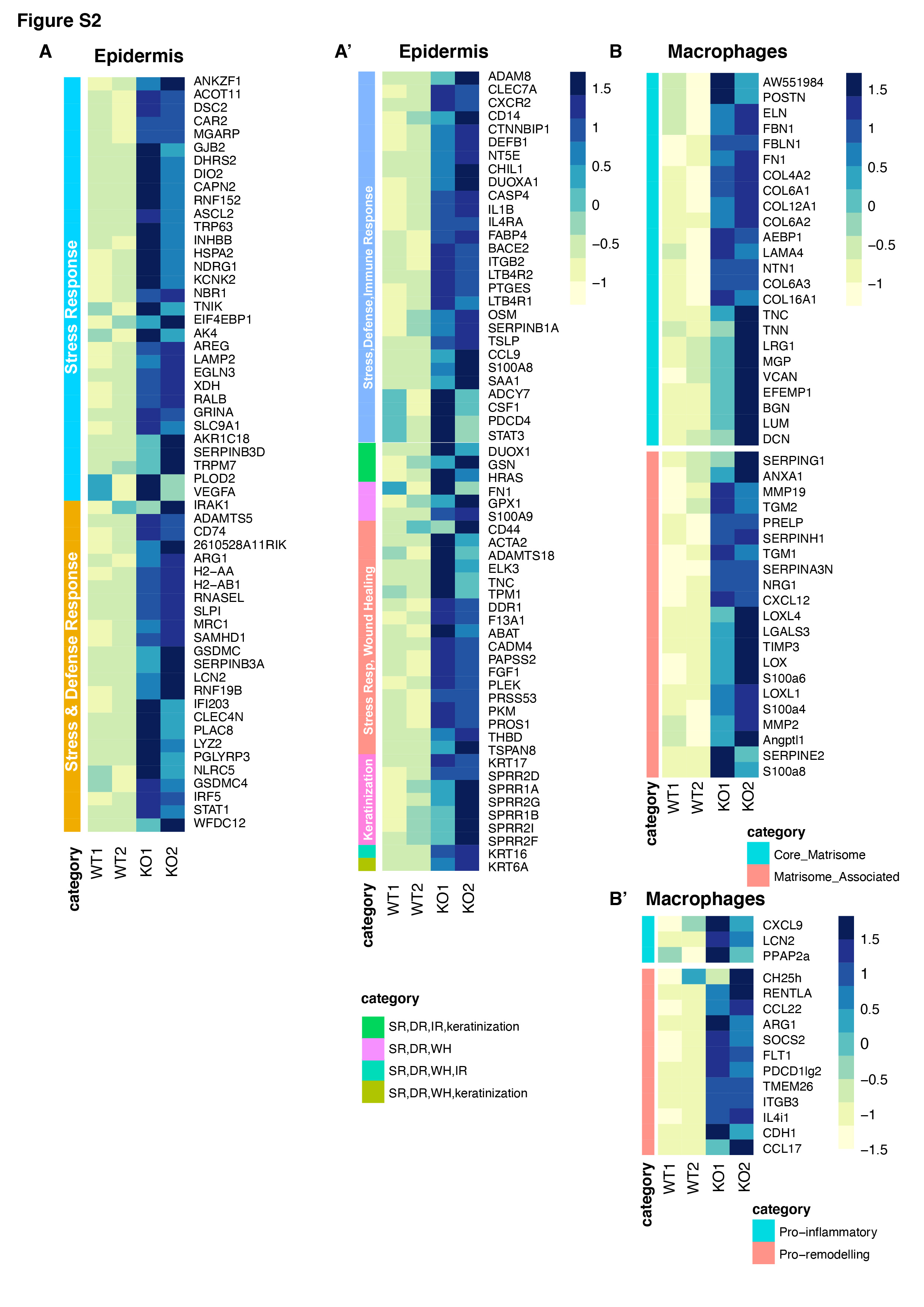

Supplement: Supplementary Figure 2 — Genes expressed in the epidermis, fibroblasts and the macrophages in the KO skin. Heat map represents the NGS analysis of the upregulated genes in the stress, defense, wound healing and the keratinization pathways in the Itgβ1 KO epidermis (A, A’). ECM transcripts upregulated in the macrophages in the Itgβ1 KO skin (B). Pro-inflammatory and the pro-remodelling associated genes in the macrophages in the epidermal Itgβ1 KO skin (B’). [file Image_2.jpeg]

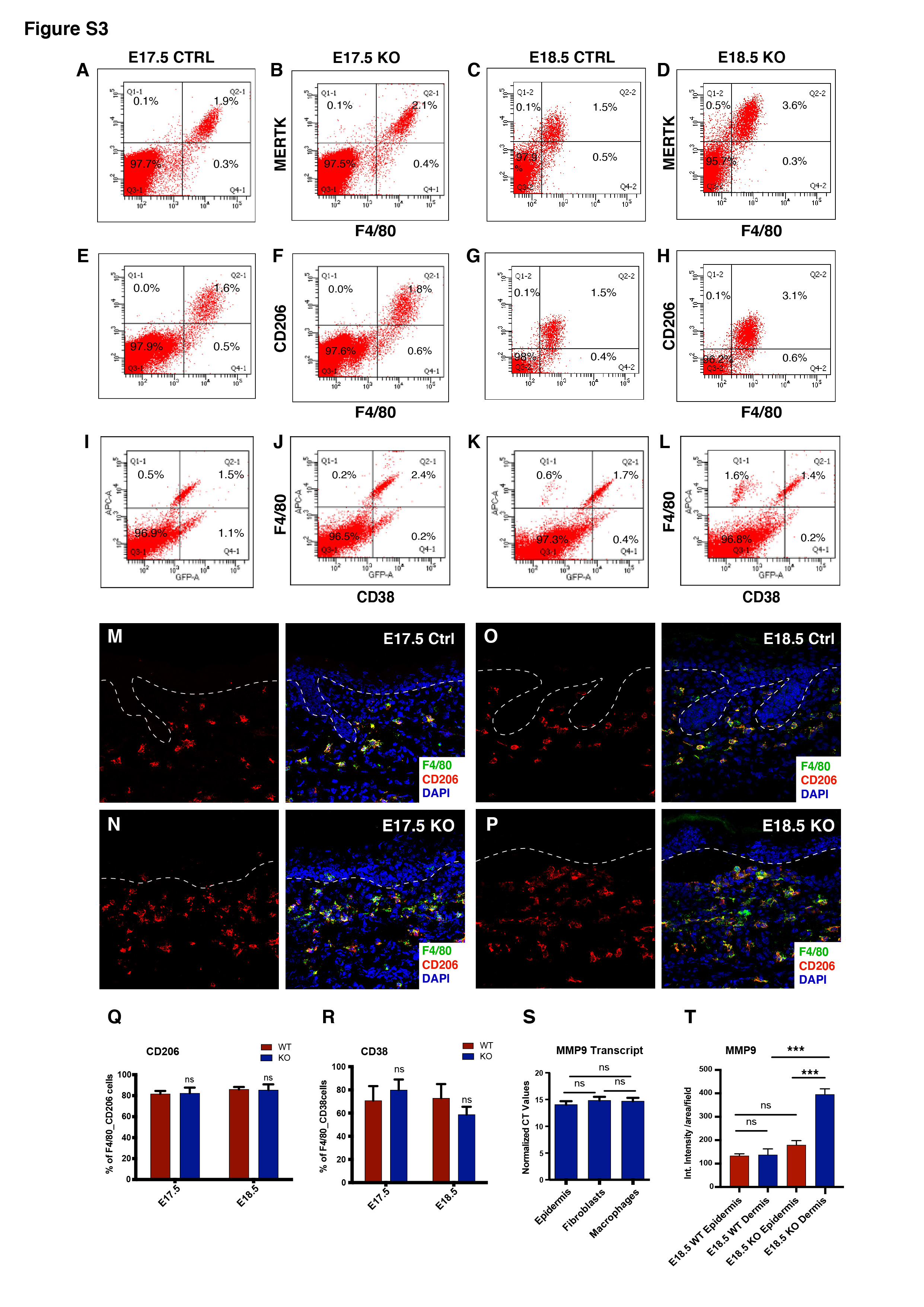

Supplement: Supplementary Figure 3 — Macrophages in the KO skin acquire exaggerated M2-like pro-remodelling properties at E17.5. Flow cytometry analysis in the E17.5, E18.5 control and Itgβ1 KO skin for the expression of F4/80 and MERTK (A–D) F4/80 and CD206 (E–H) F4/80 and CD38 (I–L). Immunostaining for F4/80 and CD206 at E17.5, E18.5 (M–P). Scale bar: 20 µm. Quantification of flow cytometry analysis for the percentage of F4/80+CD206+ and F4/80+CD38+ cell population in the skin at E17.5 and E18.5 (Q, R). Quantification of real-time PCR analysis of the MMP9 transcript in the epidermis, fibroblasts and macrophages at E18.5 (S). Quantification of the staining intensity of the MMP9 in the epidermis and the dermis at E18.5 in Control and Itgβ1 epidermal KO skin (T) (N=2; ***p≤0.001, ns=not significant). [file Image_3.jpeg]

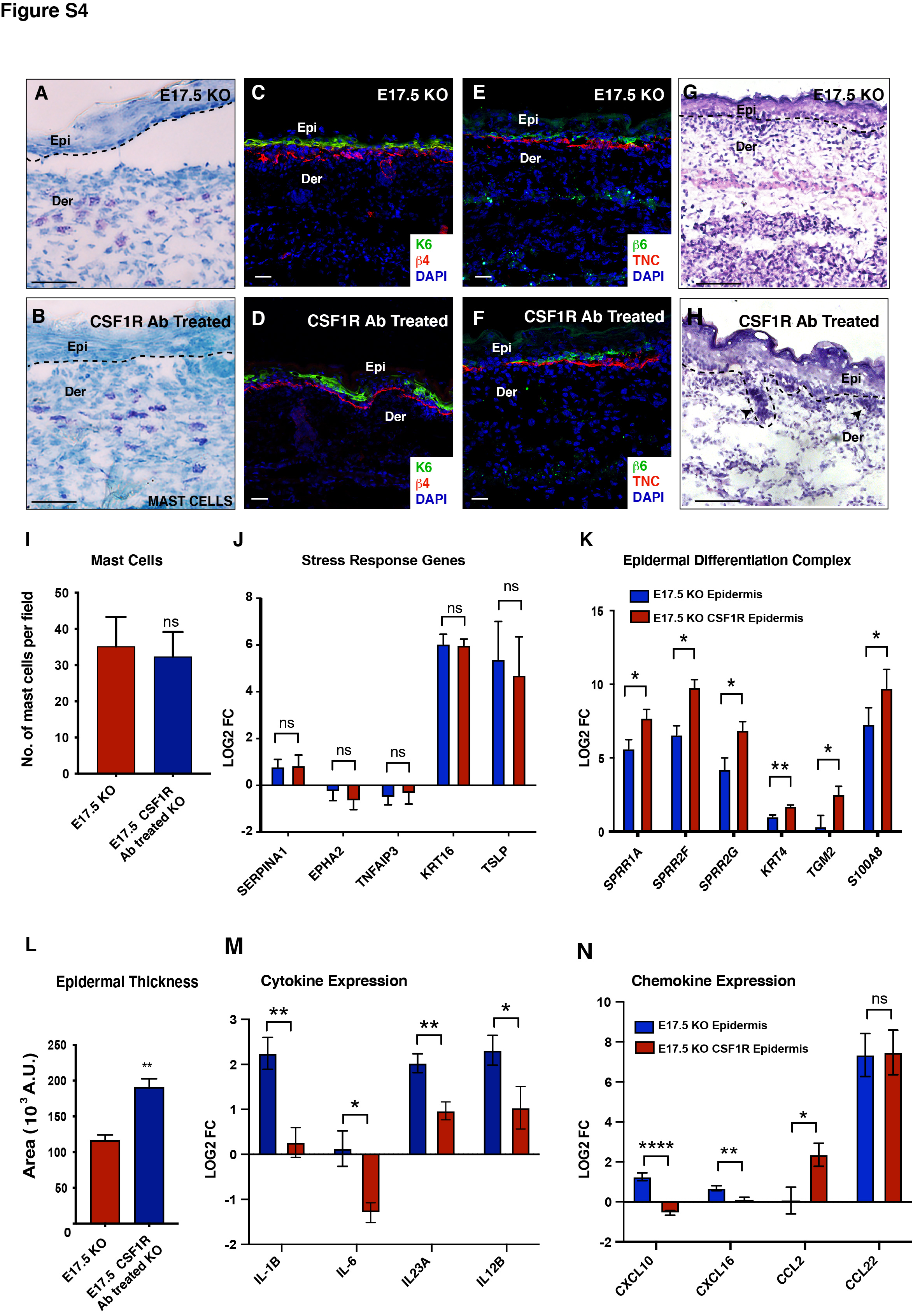

Supplement: Supplementary Figure 4 — Epidermal stress persists in the CSF1R blocked E17.5 KO skin. Toluidine blue assay for the mast cells in the PBS treated E17.5 Itgβ1 epidermal KO skin and CSF1R antibody treated skin (A, B) quantified in (I). Immunostaining for Keratin 6 (K6) and Itgβ4 (C, D); Itgβ6 and TNC (E, F) PBS treated E17.5 Itgβ1 epidermal KO skin and CSF1R blocked skin Scale bar: 20 µm. Hematoxylin and eosin staining in the PBS treated E17.5 Itgβ1 epidermal KO skin and CSF1R blocked skin (G, H). Scale bar: 50 µm. Quantification of the real-time PCR data for stress response and epidermal differentiation complex genes in the PBS treated E17.5 Itgβ1 epidermal KO skin and CSF1R antibody treated skin (J, K) (N=3; *p-value ≤ 0.05, **p-value ≤ 0.01, ns, non-significant). Quantification of the epidermal thickness in the PBS treated E17.5 Itgβ1 epidermal KO skin and CSF1R antibody treated skin (L) (N=3; **p-value<0.01). Quantification of the real-time PCR data in and CSF1R antibody treated skin for the cytokines and chemokines (M, N) (N=3; *p-value ≤ 0.05, **p-value ≤ 0.01, ****p-value≤ 0.0001 ns, non-significant). [file Image_4.jpg]

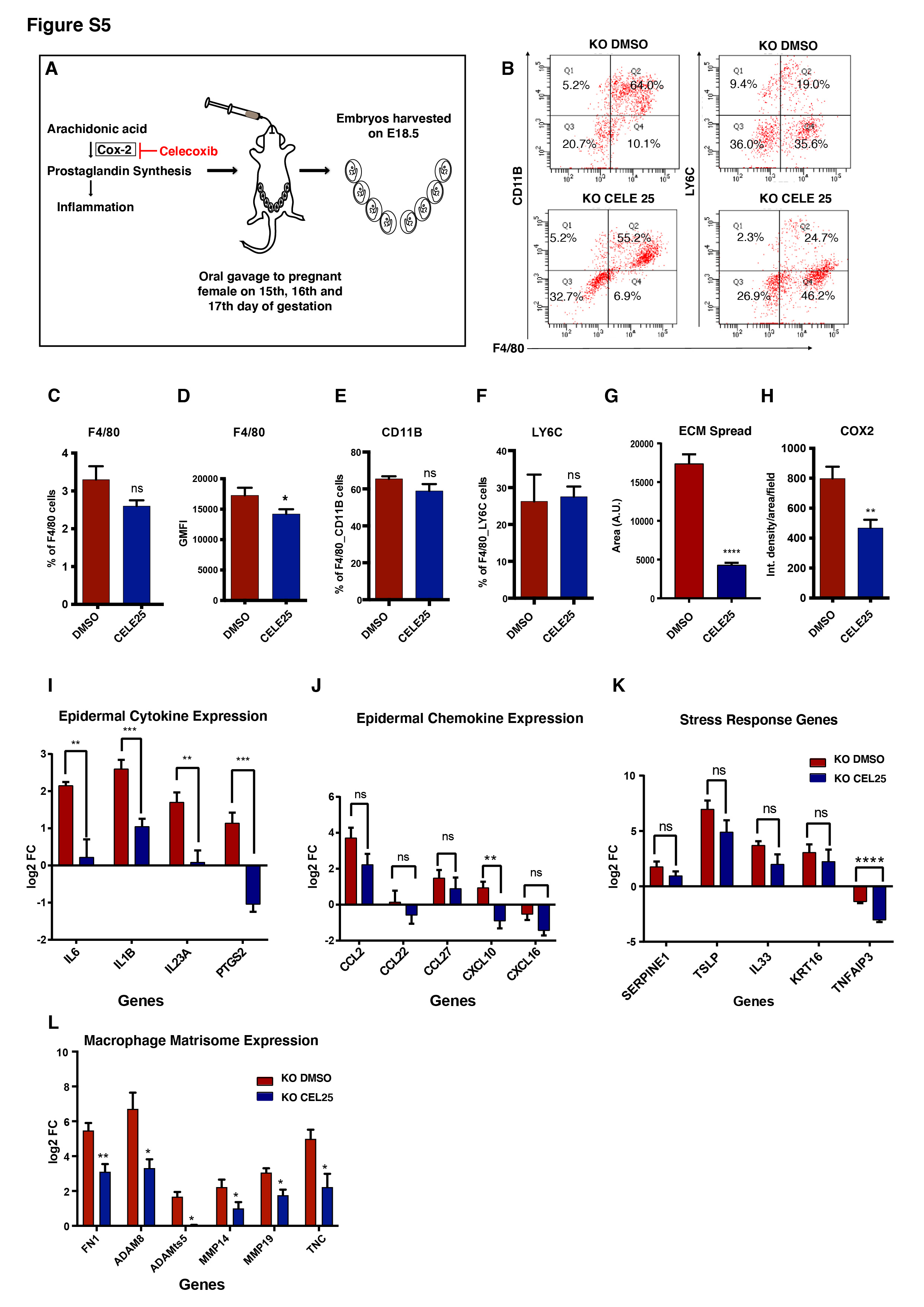

Supplement: Supplementary Figure 5 — Analysis of celecoxib treated epidermis and macrophages. The representation of the strategy of dosing celecoxib to pregnant dams (A). Flow cytometry analyses for the percentage of F4/80+, CD11B+ and LY6C+ on the CD45+ cells in the E18.5 Itgβ1 epidermal KO skin treated with DMSO and celecoxib (B). Quantification of the changes in the percentage of population of F4/80+, CD11B+ and LY6C+ cells (C, E, F) (N=2). Quantification of the surface expression of F4/80 (D) (N=2). Quantification for the ECM spread in the E18.5 Itgβ1 epidermal KO skin treated with DMSO and celecoxib (G) (N=2; ****p-value ≤ 0.0001). Quantification for the epidermal expression of Cox2 in the E18.5 Itgβ1 epidermal KO skin treated with DMSO and celecoxib (H) (N=2; ****p-value ≤ 0.001). Quantification of the real-time PCR data for the cytokines, chemokines, and stress response genes in the epidermis of E18.5 Itgβ1 KO treated with DMSO and celecoxib (I–K) (N=3; *p-value ≤ 0.05, **p-value ≤ 0.01, ****p-value≤ 0.0001 ns, non-significant). Quantification of real-time PCR analysis for matrisome transcripts in the macrophages in E18.5 Itgβ1 epidermal KO skin treated with DMSO and celecoxib (L) (N=3; *p-value ≤ 0.05). [file Image_5.jpeg]
